# Anaplastic Large Cell Lymphoma Misdiagnosed as Systemic Lupus Erythematosus: A Report of a Rare Case

**DOI:** 10.7759/cureus.44995

**Published:** 2023-09-10

**Authors:** Khadeejeh M Alfroukh, Osayd Nassar, Mosa R Abu Sabha, Zinah A Bairmani, Wala N Y. Awad, Saed I Atawnah

**Affiliations:** 1 Department of Internal Medicine, Al-Ahli Hospital, Hebron, PSE; 2 Department of Internal Medicine, Faculty of Medicine, Al-Quds University, Jerusalem, PSE; 3 Department of Pharmacology & Experimental Therapeutics, Thomas Jefferson University, Philadelphia, USA

**Keywords:** multidisciplinary collaboration, autoimmune disorders, lymphadenopathy, misdiagnosis, systemic lupus erythematosus, non-hodgkin lymphoma

## Abstract

Non-Hodgkin lymphomas are a diverse group of lymphoproliferative disorders rising from the lymphocytes with a broad spectrum of histological characteristics and clinical manifestations that often complicate accurate diagnosis. Autoantibodies have been observed at higher frequencies in lymphoproliferative diseases, yet the precise role of the immune system and the underlying causative factors remain enigmatic. Anaplastic large cell lymphoma (ALCL), an aggressive non-Hodgkin’s lymphoma variant, commonly presents in a manner akin to other aggressive lymphomas, featuring swift progression of peripheral and/or retroperitoneal adenopathy, accompanied by systemic symptoms like fever, night sweats, and weight loss. This case report delves into a histologically verified instance of ALCL, which strikingly emulates systemic lupus erythematosus. This report's objective is to underscore the concept that lymphoma can manifest clinical or biological features reminiscent of autoimmunity.

## Introduction

Systemic lupus erythematosus (SLE) is a complex autoimmune disease characterized by a wide range of clinical signs. The presence of multiple autoantibodies leads to the formation of immune complexes and triggers various immune responses [1]. Notably, SLE patients have been found to face an increased risk of malignancy, particularly hematological malignancies. Non-Hodgkin’s lymphoma (NHL) is the most prevalent malignancy [2], which is attributed to a combination of inherent immune system dysfunction and exposure to medications and viruses [3]. NHL and SLE have overlapping clinical manifestations and biological markers, including autoantibodies like antinuclear antibodies (ANA) that can pose a diagnostic challenge; thus, NHL can be misdiagnosed if the physician fails to broaden the differential diagnosis [4].

We present a case of NHL initially misdiagnosed as SLE based on the clinical symptoms including fatigue, significant weight loss, non-scarring alopecia, fever, diffuse joint pain, and oral ulcers as well as positive tests for ANA and anti-dsDNA, meeting both clinical and immunological criteria for SLE. Later, the patient was admitted to our hospital for pneumonia. A comprehensive diagnostic assessment, including physical examination, radiological imaging, and a left axillary lymph node excisional biopsy, revealed the presence of anaplastic large cell lymphoma (ALCL).

This case underscores the intriguing similarity between lymphoma and SLE presentations, highlighting the potential for lymphoma to manifest clinical or biological features associated with autoimmunity and the complexities of accurate diagnosis. 

## Case presentation

A 32-year-old married Middle Eastern woman, currently tending to her eight-month-old child was diagnosed with SLE at an outpatient clinic based on a four-month history of fatigue, fever, diffuse arthralgia, recurrent oral ulcers, skin hyperpigmentation on her hands, a non-scarring alopecia, and a profound weight loss of approximately 20 kg. She met the immunological benchmarks for SLE, with positive results for ANA and anti-dsDNA. She had no prior surgeries and no known allergies. The patient categorically denied any use of alcohol, tobacco, or illicit substances. Her family history presented no malignancies, including hematological malignancies and connective tissue diseases. She was prescribed prednisolone and azathioprine for SLE.

**Figure 1:**
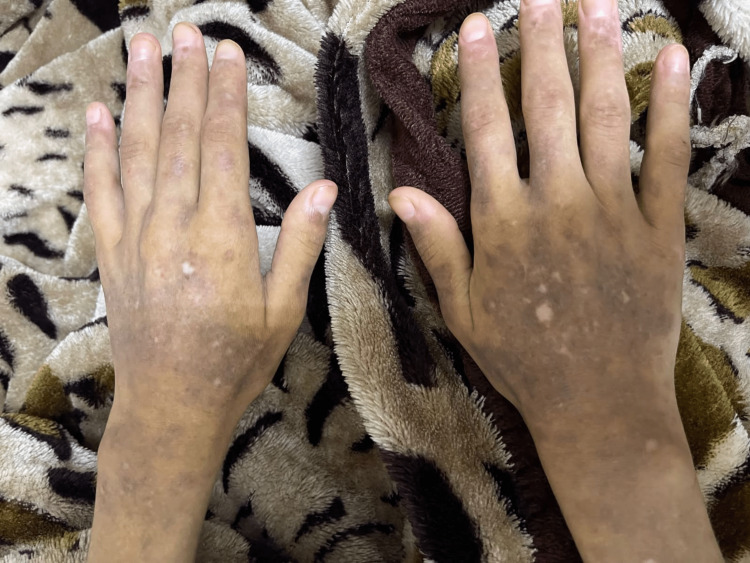
Multiple brown-to-black patches and hypopigmented macules on hands.

Two months later, the patient arrived at our hospital with a history of dry cough, progressively worsening nonexertional dyspnea, and fever. Her clinical evaluation revealed a blood pressure of 100/67 mmHg, a heart rate of 120 beats per minute, a respiratory rate of 28 breaths per minute, a temperature of 38.8°C (101.84°F), and an oxygen saturation of 87% in ambient air. Her weight was 50 kg, her height stood at 160 cm, and her body mass index was 19.5 kg/m². Despite her apparent discomfort, she remained alert, oriented, and in acute distress. Further clinical assessments illuminated tachycardia, pale conjunctiva, diminished breathing sounds in the left lower lobe, and bilateral axillary and inguinal lymph node enlargement. Also, her skin displayed multiple patches of brown-to-black macules on her hands and face, and hypopigmented macules on both her hands and feet (Figure 1).

The patient was admitted to the medical ward for comprehensive assessment and treatment. A meticulous laboratory workup was initiated, incorporating both immunological and tumor markers (Table 1).

**Table 1:** Laboratory Investigations CA 15-3: Cancer antigen 15-3; CA 19-9: Cancer antigen 19-9; CEA: Carcinoembryonic antigen

Lab Test	Result	Reference range
Hemoglobin	8.3	12-16 g/dl
Mean corpuscular volume	83.6	82-92 fL
Mean corpuscular hemoglobin	27	27-31 pg
White blood cells	7000	5000-1000/mm^3^
Neutrophils	7300	2500-7500/ul
Lymphocyte	650	1500-3500/ul
Platelet	197	150000-400000/mm^3^
Erythrocyte sedimentation rate	100	0-20 mm/h
C-reactive protein	101	Up to 6 mg/L
Antinuclear antibodies (ANA)	4.6	< 0.8
Anti-Ds DNA IgG	73.4	< 20 IU/ml
Anti- Ds DNA IgM	145.7	< 20 IU/ml
Anti-Smith	Negative	Negative
C3	56	80-176 mg/dl
C4	11.5	15-57 mg/dl
Alpha-fetoprotein	1.03	Up to 10.9 ng/ml
CA 15-3	21.7	0-31.3U/ml
CA 19-9	2.07	0-37U/ml
CEA	1.25	Non-smoker 0-3ng/ml, Smoker 0-5ng/ml

**Figure 2:**
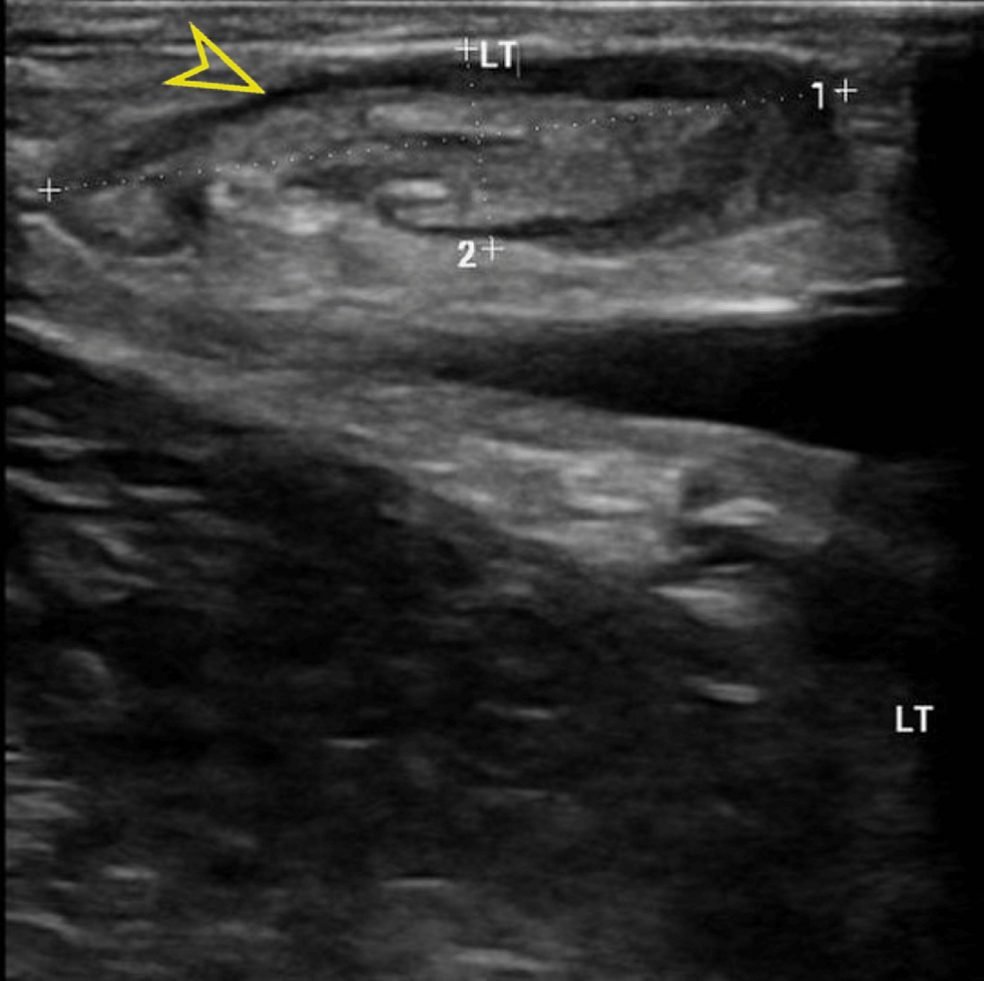
Correlated Inguinal ultrasound shows multiple prominent bilateral oval-shaped inguinal lymph nodes with preserved fatty hilum.

**Figure 3:**
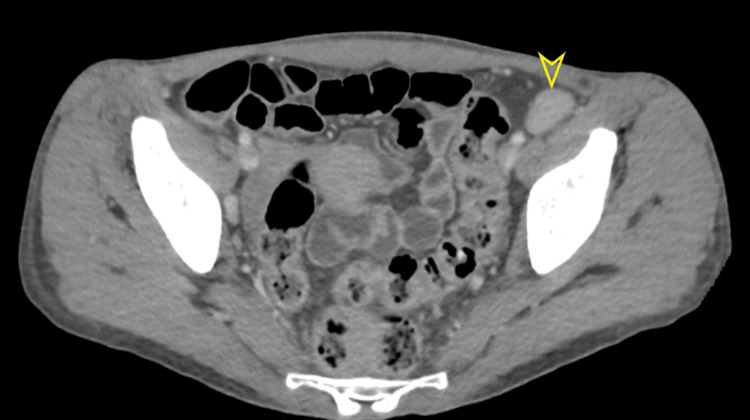
Selected image of axial contrast-enhanced abdominal CT scan showing multiple enlarged pelvic lymph nodes, the largest measures about 24 x 14 mm noted at the left external iliac group.

**Figure 4:**
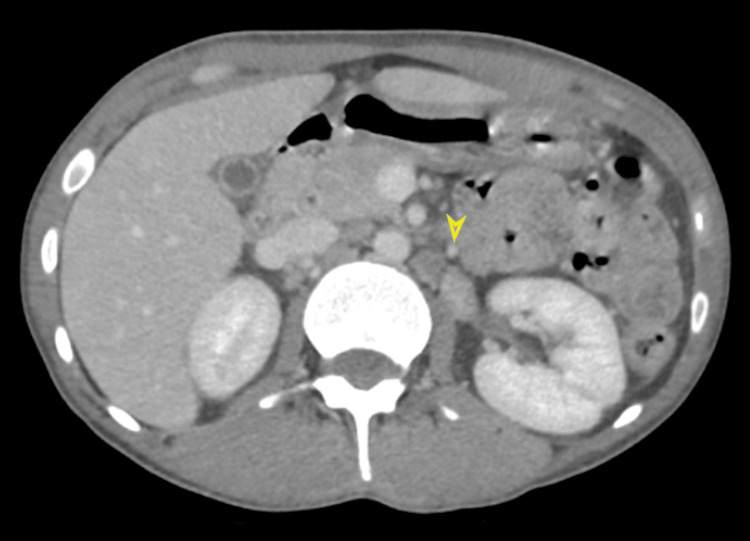
Selected cut of enhanced axial abdominal CT scan showing multiple enlarged para-aortic lymph nodes, the largest measures about 19 x 12 mm noted at the left para-aortic group at the level of the left kidney hilum.

**Figure 5:**
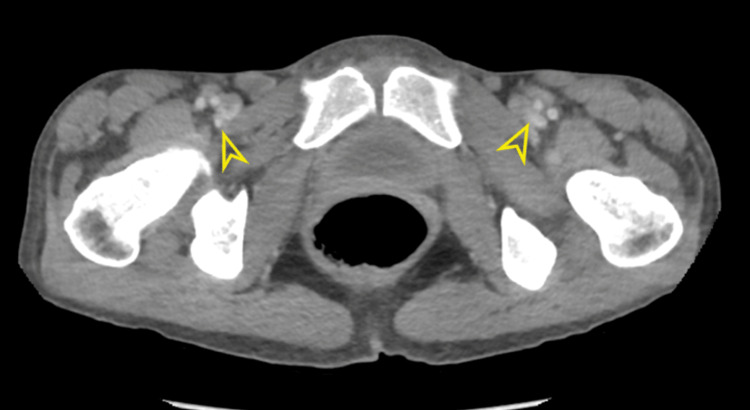
Selected image of axial contrast-enhanced abdominal CT scan showing multiple prominent bilateral inguinal lymph nodes.

Subsequent ultrasound examinations of the neck, breast, and inguinal regions revealed the presence of enlarged intra-parotid lymph nodes, bilateral axillary lymph nodes enlargement, and substantial bilateral inguinal lymph nodes (Figure 2). Computed tomography (CT) scan of the chest, abdomen, and pelvis with IV contrast was conducted to identify potential intra-abdominal masses or lymph nodes. The scan revealed left lower lobe consolidation with an air-bronchogram indicative of lobar pneumonia. Furthermore, it identified mild bilateral pleural effusion accompanied by scattered ground glass opacities and tree-in-bud nodules, suggestive of an atypical infection. Cardiomegaly with minimal pericardial effusion was also noted. Multiple pelvic (Figure 3), para-aortic (Figure 4), and inguinal lymph nodes were notably enlarged (Figure 5). 

Driven by these investigative outcomes, a left axillary lymph node biopsy and skin biopsy for the hyperpigmented skin lesions were performed. Throughout her hospital stay as a case of SLE flare and community-acquired pneumonia, her treatment regimen encompassed azathioprine, prednisone, and ceftriaxone. After a six-day hospitalization period, she was discharged with prescriptions for hydroxychloroquine (200 mg once daily) and prednisone (20 mg once daily). 

**Figure 6:**
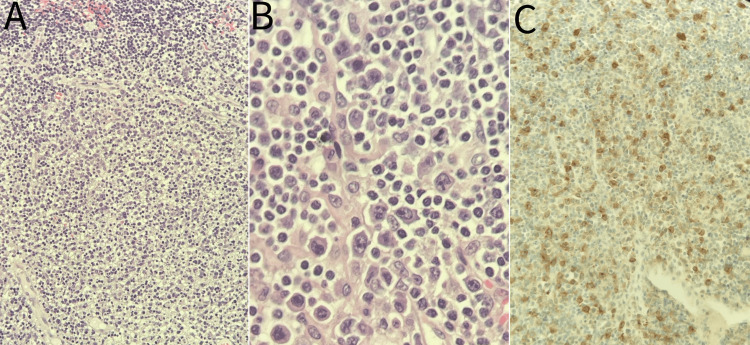
(A) Monomorphic to pleomorphic large lymphocytes with prominent nucleoli and abundant eosinophilic cytoplasm (H&E: 20x); (B)Tumor infiltration of lymph node sinuses. Some of the large cells have horseshoe or doughnut-shaped nuclei (hallmark cells) (H&E: 40x); (C) Diffusely positive CD30 immunostaining. H&E: hematoxylin and eosin; CD: cluster of differentiation

One week later, the skin biopsy outcome indicated pigment incontinence with melanin lying free in the superficial dermis and the accumulation of pigmented melanophages in the superficial dermis. The lymph node biopsy finding was consistent with ALCL (Figure 6). In this context, ALCL emerged as the underlying explanatory factor for all clinical and immunological manifestations.

The patient was recommended for an anaplastic lymphoma kinase (ALK) protein test, a diagnostic test not currently accessible within our medical facility. Additionally, the attending oncologist was poised to initiate a course of treatment involving brentuximab vedotin, cyclophosphamide, doxorubicin, and prednisone as a part of the induction therapy protocol.

## Discussion

We presented a challenging case of a 32-year-old female who was diagnosed with ALCL that mimicked the clinical and immunological criteria of SLE based on the 2019 European League Against Rheumatism/American College of Rheumatology (EULAR/ACR) classification criteria.

SLE has been linked to an elevated risk of lymphoma, particularly NHL. A comprehensive review of the existing literature, encompassing multiple studies, consistently affirms the association between SLE and lymphoma development. This association typically becomes evident several years following the initial SLE diagnosis, particularly among individuals undergoing immunosuppressive medication regimens [5]. Moreover, notable research findings contribute to this understanding. A cohort study conducted in 2005 reported a median age of lymphoma occurrence in SLE patients at 57 years [6]. Another cohort study indicates that lymphoma tends to manifest, on average, approximately 12.4 years after the diagnosis of SLE (median= 10.9) [7].

In our case, it is noteworthy that the reported symptoms occurred within a relatively short timeframe, and their collective presentation aligns with a plausible explanation rooted in lymphoma. Additionally, in accordance with the 2019 EULAR/ACR classification criteria for SLE, it is essential to recognize the guiding principle that when a more plausible explanation exists for clinical findings, certain criteria should not be regarded as definitive [1]. Collectively, these findings strongly suggest that the underlying condition in this patient may be attributed to lymphoma.

Anti-dsDNA is a recognized biomarker primarily associated with SLE. However, it is noteworthy that its positivity can also be observed in various other clinical conditions, such as infections and malignancies. In a retrospective study conducted in 2010 involving 212 patients, the findings indicated that anti-dsDNA was detected as positive in 41.5% of cases unrelated to SLE [8]. Additionally, compelling evidence from case reports further substantiates the occurrence of anti-dsDNA in malignancies such as lung adenocarcinoma, and prostate cancer where the presentation of anti-dsDNA antibodies can mimic SLE but, in reality, is linked to the underlying malignancy [9,10]. Furthermore, it is important to acknowledge that certain NHLs exhibit the capacity to produce autoantibodies, emphasizing the complexity of autoantibody generation in various medical contexts [11].

Many cases of lymphoma presented with features of SLE such as non-scarring alopecia, oral ulceration, and skin hyperpigmentation [12-14]. Although the underlying mechanism for this overlapping between autoimmune diseases and lymphoproliferative disorders is still unclear, environmental, and genetic factors may play a role [15]. 

Finally, relying solely on serological markers for diagnosis can lead to misdiagnosis. As a result, this case emphasizes the need to incorporate a detailed history and physical examination, diagnostic imaging, and histopathological examination. A biopsy is crucial to differentiate between autoimmune diseases and lymphoproliferative disorders. Also, this case report emphasizes the need for multidisciplinary collaboration between rheumatologists, pathologists, and oncologists to accurately diagnose malignancies presenting as autoimmune-like manifestations.

## Conclusions

This case of ALCL initially misdiagnosed as SLE clarifies the significance of considering lymphoma in the differential diagnosis of autoimmune diseases, and multidisciplinary collaboration to ensure accurate diagnoses and initiation of appropriate treatment. The intricate interplay between these conditions underscores the profound ability of lymphomas to mimic autoimmune disease, imparting clinical and immunological traits. Finally, by Increasing awareness of the clinical overlap between ALCL and SLE, clinicians can avoid misdiagnosis.
